# ROS-Influenced Regulatory Cross-Talk With Wnt Signaling Pathway During Perinatal Development

**DOI:** 10.3389/fmolb.2022.889719

**Published:** 2022-04-19

**Authors:** Sharmistha Chatterjee, Parames C. Sil

**Affiliations:** Division of Molecular Medicine, Bose Institute, Kolkata, India

**Keywords:** wnt, ROS, cross-talks, signaling, perinatal development, embryogenesis

## Abstract

Over a century ago, it was found that a rapid burst of oxygen is needed and produced by the sea urchin oocyte to activate fertilization and block polyspermy. Since then, scientific research has taken strides to establish that Reactive Oxygen Species (ROS), besides being toxic effectors of cellular damage and death, also act as molecular messengers in important developmental signaling cascades, thereby modulating them. Wnt signaling pathway is one such developmental pathway, which has significant effects on growth, proliferation, and differentiation of cells at the earliest embryonic stages of an organism, apart from being significant role-players in the instances of cellular transformation and cancer when this tightly-regulated system encounters aberrations. In this review, we discuss more about the Wnt and ROS signaling pathways, how they function, what roles they play overall in animals, and mostly about how these two major signaling systems cross paths and interplay in mediating major cellular signals and executing the predestined changes during the perinatal condition, in a systematic manner.

## Introduction

Evolution of living beings on earth has occurred over millions of years by achieving higher degrees of complexities in their bodily structure and functions, and degrees of organization of cells and tissues, to suit the changing environments and adapt to their niches (Darwin and Wallace 1858) (Aldhebiani 2018). When we look at the underlying systems owing to which the living organisms successfully took over the earth and its resources, we find that there was a common basis to all of them–communication–between cells and their immediate environment, be it biotic (cell to cell), or abiotic (cells responding to abiotic factors) (Thannickal and Fanburg 2000). All kinds of physical, chemical or biological stimuli evoke responses from the recipient cells *via* certain organic and inorganic mediators, which communicate the signals received by the cells to other molecules, in order to generate a response. In multicellular organisms, this mediating system or cell signaling system as we know it, is much more elaborate and extensive as compared to the unicellular organisms, as the multicellular organisms are themselves able to function efficiently as a whole, owing to the intricate communication mediated by the innumerable molecules inter-playing and reprising their roles to achieve even the most basic day to day functions like respiration, growth, proliferation, differentiation, inflammation, wound healing, and death. For higher organisms with organ system levels of organization, this signaling system branches and broadens into highly efficient networks with positive and negative feedback loops, keeping themselves tightly regulated within a species-specific framework (Marijuán et al., 2013) (Loh et al., 2016).

**FIGURE 1:**
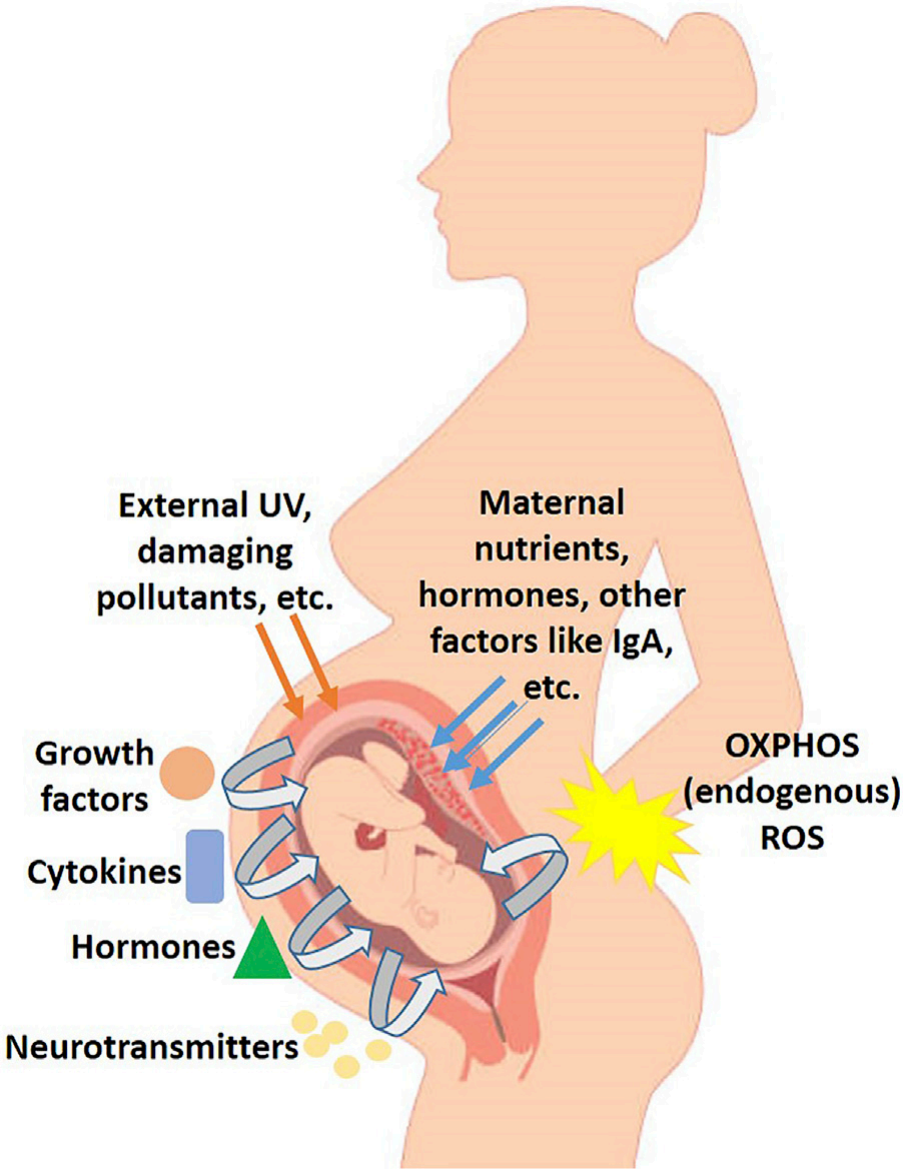
Schematic diagram of the various factors and signaling involved in development of the embryo *in utero*.

Hence, every multicellular organism depends on highly elaborate and complex signaling networks composed of extracellular as well as intracellular signals to efficiently put together cell to cell communication in varying physiological processes, beginning right from developmental organogenesis, moving on to tissue homeostasis and maintenance of normalcy in diverse physiological parameters, to healing wounds and mounting repair and inflammatory responses to tissue injuries (Thannickal and Fanburg 2000). In higher animals, when we look at the cellular levels, the extracellular signals can generally be classified into growth factors, hormones, cytokines, and neurotransmitters, which evoke certain responses from the recipient cells (Figure 1). They first bind to their specific receptors on the cellular surface, and these ligand-receptor interactions subsequently go on to generate varying intracellular signals which may manifest as trimeric GTP-binding regulatory proteins getting activated (G protein-coupled receptors, GPCRs), intracellular ion concentrations getting changed (ion channel-linked receptors), or receptor kinases getting activated (enzyme-linked receptors, like receptor tyrosine kinases, RTKs). Certain second messengers (like cAMP, Ca^2+^, and phospholipid metabolites) and/or cascades of protein phosphorylation then relay the downstream signaling further. Finally, these intracellular signaling pathways evoke the expected response by going on to activate one or multiple transcription factors that would regulate specific sets of genetic expression, which are essential for an array of cellular functions (Bhalla 2004).

One of the basic regulatory signaling pathways that has immense effects on an organism’s ontogeny, growth, development, differentiation, and all the other processes involved in fetal development, as well as adult cellular growth and tissue homeostasis, is the Wnt signaling pathway (Logan and Nusse 2004) (Gupta et al., 2016). Secreted glycoproteins by nature, Wnts are some of the most essential molecules for the animal development process. Across the animal kingdom, these proteins use various signaling pathways to play varied roles in crucial developmental processes like pattern formation during embryogenesis, to very specific and intricate processes like cell proliferation and differentiation (Clevers 2006). In vertebrates, they play very specific roles in cell fate determination, apoptosis, proliferation, and self-renewal. Very naturally, for this reason, they have been intensely researched for their roles, and found to be a key role-player in cancer development (Polakis 2000) and maintenance (Chatterjee and Sil 2019), osteoarthritis (Corr 2008) and bone density regulation (Maeda et al., 2019), and even neurodegenerative diseases (Libro et al., 2016), which all have prolonged causation periods and definitely encompass deregulated growth and apoptotic processes (Komiya and Habas 2008).

With the advent of oxygen in earth’s atmosphere and the subsequent evaluation of aerobic organisms, molecular oxygen became essential for the basic survival of all those aerobic beings (Hsia et al., 2013). Oxidative phosphorylation (OXPHOS) process mediates the conversion of reducing equivalents from FADH2 and NADH of the electron transport chain occurring in the mitochondria into the high-energy phosphate bond contained in ATP, the energy currency of the aerobic cell. This OXPHOS process generates many partially reduced, highly reactive O_2_ metabolites, including the superoxide anion and hydrogen peroxide, and in some instances of already accumulating oxidative stress, the hydrogen peroxide molecule is not fully reduced by the endogenous catalases, and becomes reduced by intracellular iron, donating an electron, in a Fenton-type reaction, to form the even more highly-reactive hydroxyl radical (Thomas et al., 2009). These highly reactive, partially reduced O_2_ metabolites are collectively termed as reactive oxygen species, or ROS. For decades, ROS have been thought of only as toxic by-products of aerobic metabolism, that potentially damage proteins, lipids and DNA (Auten and Davis 2009). This traditional thought was substantiated with the discoveries of natural antioxidant enzymes produced inside the body, like catalase, superoxide dismutase, and peroxidases, that protect the body from damaging, and often lethal effects of very high levels of ROS in the system, a condition termed as “oxidative stress”. Oxidative stress thus occurs when the system of check and balance between the production of ROS and the antioxidant enzyme activity countered an imbalance, and the scale tipped towards the production of copious amounts of ROS in the body (Sies 1997; Sinha et al., 2013). This has been shown to lead to a number of deadly diseases and debilitating conditions (Auten and Davis 2009) like cancer (Dayem et al., 2010; Kryston et al., 2011; Gurer-Orhan et al., 2018), fibrosis (Zhao et al., 2008; Gutiérrez-Tenorio et al., 2017; Jie et al., 2019; Tucker et al., 2019), neurodegenerative diseases (Kim et al., 2015) and aging (Bokov et al., 2004; Davalli et al., 2016; Peters et al., 2020).

### 1.1 Generation and Propagation of ROS in the Body

ROS can be generated by a host of environmental pollutants and generic sources which also include ultraviolet radiation of the Sun, and other ionizing pollutants like paraquat (or methyl viologen), that go on to react, being highly reactive in nature, to form ozone and other peroxides. They also react to and support the formation of chemicals promoting formation of other hyper-reactive oxidants like various nitroaromatics, quinones, and other pollutants like bipyrimidiulium herbicides that are related to paraquat. ROS also participate in chain reactions of chemical formations that are again subsequently metabolized to radicals, like the phenols, aminophenols, polyhalogenated alkanes or other chemicals, that in their lifetime do release copper and iron, that could support and promote the formation of hydroxyl radicals in future. Rapid radiolysis of water molecules upon exposure to ionizing radiation like X-rays or ᵧ-rays, or even UV irradiation of H_2_O_2_ can also generate ROS. In addition to all of these, the presence of any UV-radiation sensitizer can trigger the formation of super-reactive chain reaction inducer and moderator, singlet oxygen molecules. Non-DNA reactive carcinogens can also generate ROS in cells by metabolism, by either activating endogenous ROS sources, or by metabolizing to primary radical intermediates. Chemicals that induce and prolong oxidative stress in cells are varied–different classes of chlorinated compounds, metal ions, phorbol esters and other peroxisome-proliferating compounds, including barbiturates. The classic antitumor drugs cisplatin and Adriamycin have been shown to produce ROS at excessive levels intracellularly, resulting in DNA damage and cell death. Some classes of antibiotics rely on a similar mechanism for their bactericidal activity, which incorporate Fenton-type reactions for their bactericidal activity (Krumova and Cosa, 2016).

In living organisms, cytosolic enzymes that contribute to the generation and propagation of ROS, including the seven isoforms of the ever-expanding family of NADPH oxidases (NOxs), the ETC transmembrane super-oxide generating system, contribute to the generation of ROS. The cytosolic portions of these enzymes transfer an electron from the NADPHs to an FAD co-factor, the electron is thereby passed-on to the haem group, subsequently being added to molecular oxygen, generating superoxide anion O_2_˙^−^. Mitochondrial sources add on important sources of ROS as additives. Antioxidant enzyme systems work in the body at all times, each and every second in the body, to avoid the generation of oxidative stress, which happens when the delicate balance of pro-oxidant and anti-oxidant system in the body is affected, by exogenous or endogenous means (Ziech et al., 2010).

Incomplete reduction of oxygen in the electron transport chain produce ROS, i.e., Reactive oxygen species (ROS), the category of highly active radicals including hydrogen peroxide (H_2_O_2_), the hydroxyl radical (^**.**^OH), the superoxide anion (O_2_^−^), and peroxynitrites (ONOO^−^), which are all are chemically active, in many cases exogenous, prooxidant molecules that are generated by various physical and chemical redox processes. ROS are involved in a variety of physiological and pathological processes in the cell by generating various methods of cellular survival and glycolysis (e.g., anaerobic glycolysis). It has been established that cancer cells continue to thrive under some levels of oxidative stress, and compared with non-cancerous tissues, ROS levels are elevated in breast, colon, pancreatic, prostate, and other cancers (Pavlides et al., 2009). In addition, oxidative DNA damage by ROS contributes to carcinogenesis by causing mutations in the genetic material. ROS in addition, can cause single-strand breaks and genetic instability, and this is due to oxidation of purines and pyrimidines and generation of alkali-labile sites. Oxidative damage that commonly leads to mutations is associated with modification of Guanine-Cytosine (G-C) base pairs, and these mutations are primarily due to base pair substitution and sometimes, also due to (albeit, less frequent) deletions and insertions (Lin et al., 2018).

### 1.2 Other Roles of ROS

But then more and more evidence on other fronts started accumulating in favor of these very same ROS acting as important mediators in the cell signaling networks and regulation of physiological homeostasis (Thannickal and Fanburg 2000). There has been increasing research in this regard since the discovery of OxyR proteins functioning as transcriptional regulators for H_2_O_2_ inducible genes in *E. Coli*, and since then, the role of ROS as important cell signaling messengers have been documented in plant cells where H_2_O_2_ is generated to effect localized cell death in response to pathogenic attack, which limits the pathogenic spread in the plant body. ROS has also been found to effect systemic responses in inducing defense genes that regulate plant immunity. All the phagocytic cells contain plasma membrane oxidases that generate O_2_^2-^(peroxide ion), and act in host defense mechanisms by producing copious amounts of ROS. Fertilization event in sea urchins also triggers extracellular H_2_O_2_ production that catalyzes extracellular protein cross-linking to form a protective fertilization envelope around the oocyte (Shapiro 1991; Wong et al., 2004). All these evidences indicated that just like nitric oxides, ROS might have dual effects as regulators and cytotoxic agents, depending upon their source and concentration (Finkel 2011).

In this extensive review, taking cues from the documented effects of Wnts in animal development and growth, as well as the role of ROS as important mediators of cellular signaling even in oocytes, we would primarily focus on how the developmental Wnt signaling might be affected by ROS and vice-versa, during the perinatal period (Table 1). Tight regulation of extracellular and intracellular signals and molecular homeostasis during the perinatal period is especially essential for the foetus to grow into healthy individuals and in a wider sense, the sustenance of species, and any minor dysregulation might lead to lethality, and debilitating developmental defects (Gilbert 2000). Therefore, for better monitoring of fetal and neonatal health, and to further elucidate the varied roles of ROS other than mere toxic by-products, we choose the major developmental pathway Wnt, to be shed light upon. This review thus covers the specifics and nuances of Wnt signaling, modes of mediation and effects of ROS in the process, and also brushes across the effects that might be observed if this highly regulated system gets broken.

**TABLE 1:** Table of contents of review.

Section number	Name	Content
1	Introduction	Basic background of the review
1.1	Generation and propagation of ROS in body	Exogenous and endogenous sources of ROS
1.2	Other roles of ROS	Beneficial, and other roles of ROS in the body
1.3	Wnt signalling pathway	Role and function of Wnt signalling pathway in body
1.4	ROS-mediated signalling pathways	How ROS work in signalling systems in the body
2	ROS-Wnt interplay in developmental signalling networks	Discussions of Wnt-ROS interplays in perinatal signalling
2.1	General mechanistic aspects	General mechanistic aspects of ROS-Wnt interplay
2.2	Specific interactions and roles	Organ and organ system-specific developmental signalling interplays of ROS and Wnt
2.2.i	Cell death and apoptosis	Discussion
2.2.ii	Embryological patterning	Discussion
2.2.iii	Lung development, differentiation, and damage	Discussion
2.2.iv	Neural development and differentiation, senescence and neuronal damage	Discussion
2.2.v	HSC sustenance and differentiation	Discussion
2.2.vi	Vascular differentiation	Discussion
2.2.vii	Nephrological development	Discussion
2.2.viii	Extraembryonic endoderm formation and differentiation	Discussion
2.2.ix	Cardiac differentiation and protection	Discussion
2.2.x	Fibrogenic differentiation: Osteogenesis, adipogenesis, and chondrogenesis	Discussion
2.2.xi	Cellular senescence	Discussion

### 1.3 Wnt Signaling Pathway

The Wnt signaling pathway is an evolutionarily conserved cellular communication pathway that determines cell polarity and fate, embryo patterning, as well as self-renewal, that is, origin and maintenance of stem cells. Two other major signaling cascades work in tandem with Wnt during embryological development, them being Notch and Hedgehog, which are also functional in stem cell growth and maintenance. Originally, the Wnt protein was discovered in *Drosophila* as the product of wingless gene (orthologous form), and then, research found the Wnt family of genes to be highly conserved in the animal kingdom, starting from nematodes to mammals (Funato et al., 2006). Being one among the key gene families which determine patterning during embryonic development, in vertebrates alone, the Wnt genes are known to consist of 19 distinct members, and all of them encode proteins that take one of the three known routes for signaling–the canonical Wnt pathway involving β-catenin (Wnt/β-catenin), or the noncanonical planar cell polarity (PCP) pathway involving cJun N-terminal Kinase (also called Wnt/JNK-PCP pathway), or the other noncanonical Wnt pathway involving Ca^2+^ (Wnt/Ca^2+^ pathway) (Angers and Moon 2009; van Amerongen and Nusse 2009).

**FIGURE 2:**
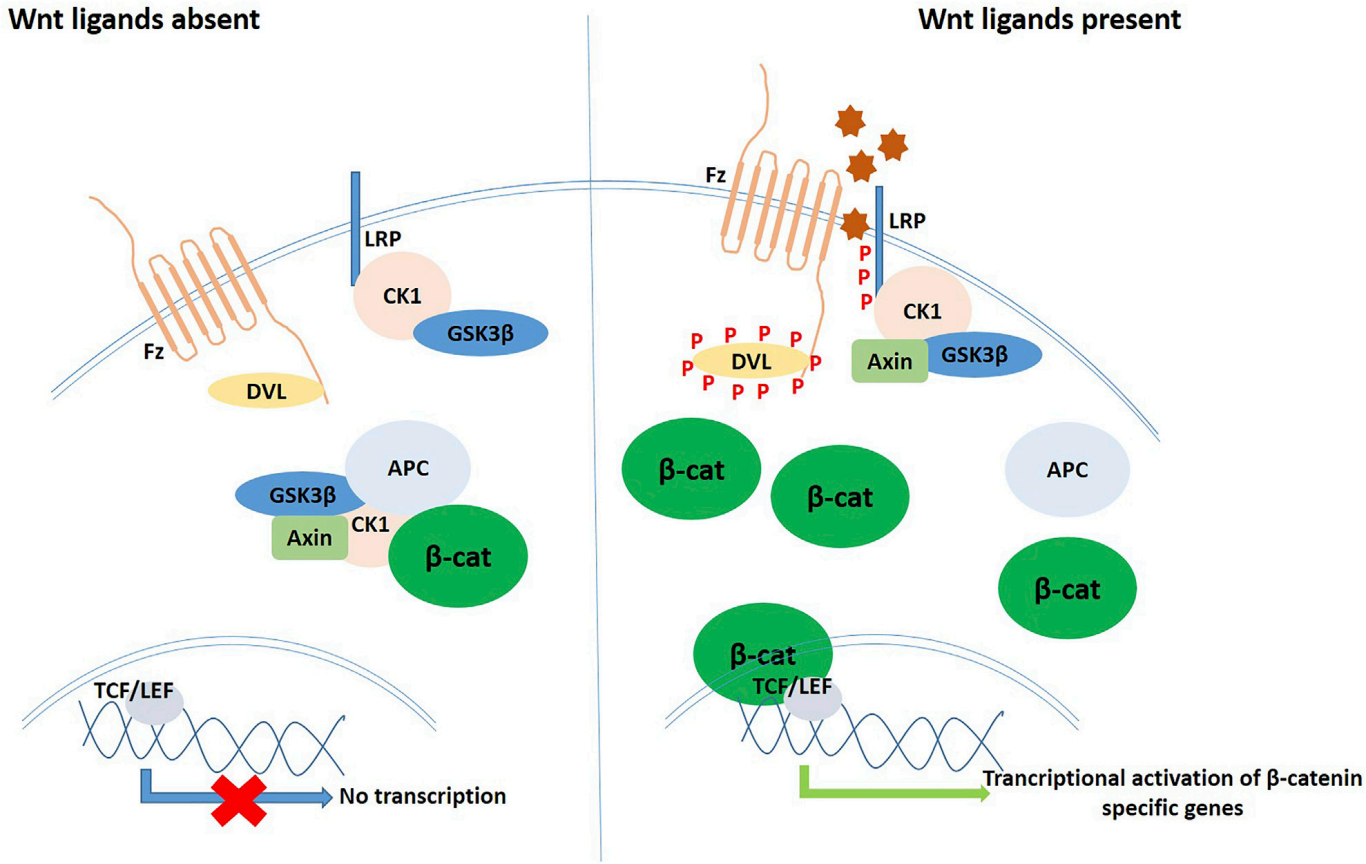
Schematic diagram of the ROS signalling cascade in operation *via* different modes and pathways.

The Wnt glycoprotein homologues are secreted out of the cells *via* Wntless, a specialized transmembrane protein, and act as ligands for Frizzled (Fz), a G-protein coupled 7-pass transmembrane receptor. All of them bind to a cysteine-rich conserved region of Fz thus activating it, and in the canonical mode, evoke a cellular response by inhibiting the β-catenin degradation complex, that is composed of four proteins–Axin, Adenomatous Polyposis Coli (APC), GSK3β, and casein kinase 1 (CK1), thereby stabilizing β-catenin in the cytosol. The accumulated β-catenin then migrates to the nucleus to interact with T-cell factor/Lymphocyte enhancer factor (TCF/LEF) family of transcription factors, thereby activating the transcription of target genes (Figure 2). A major component of this degradation complex inhibition, once the Wnt ligand has bound to Fz, is Dishevelled (Dvl)—a protein that we would come across many times in this literature, that has been found to be one of the key role players of ROS-mediated Wnt activity (van Amerongen and Nusse 2009). Before secretion from the cell, the Wnts undergo additional biochemical modification so that the LRP (Lipoprotein-receptor related protein) 5/6 receptors, which are the co-receptors mediating canonical Wnt signaling, get properly internalized (Takada et al., 2006; Komekado et al., 2007). The specific cellular responses that are elicited by the binding of different Wnt homologues are attributed to the differential binding propensity of the Wnt ligands to the highly conserved cysteine-rich region of the Fz receptor (Schulte and Bryja 2007). The Ca^2+^ responsive non-canonical pathways are independent of β-catenin, and besides the usual transcription activation responses, they also evoke cytoskeletal responses mediated by JNK and Rho-associated protein kinase (ROCK) (Polakis 2000). During embryogenesis of higher animals, the Wnt pathway directs the development of nearly all the major organs and organ systems (Eberhart and Argani 2001), including cardiovascular system, the central nervous system, respiratory as well as the excretory (renal) system (Grigoryan et al., 2008; Taciak et al., 2018).

### 1.4 ROS -Mediated Signaling Pathways

It is a well-established fact now that oxidative stress targets receptor kinases and phosphatases and deregulates their functioning and the signaling pathways mediated by them (Wu 2004; Matés et al., 2008; Gaki and Papavassiliou 2014). Even though historically, these ROS have been purely viewed as harmful and toxic, leading to cellular damage, ample evidence now exists to ascertain the fact that they also function as essential physiological modulators and regulators of multiple intracellular signaling pathways (Griendling et al., 2000; Fialkow et al., 2007; Schönenberger and Kovacs 2015; Sies and Jones 2020). These ROS-specific effects mainly occur *via* covalent modification of redox-sensitive target proteins, at some of their specific cysteine residues. Enzymatic activities of these proteins can be reversibly modified by the oxidation of these reactive and very specific cysteine residues. Multiple emerging evidence now suggests that ROS might regulate an array of physiological parameters ranging from growth factor stimulation responses to inflammatory response generation, and very obviously, deregulations in these ROS signaling pathways may contribute to a multitude of human diseases (Finkel 2011).

**FIGURE 3:**
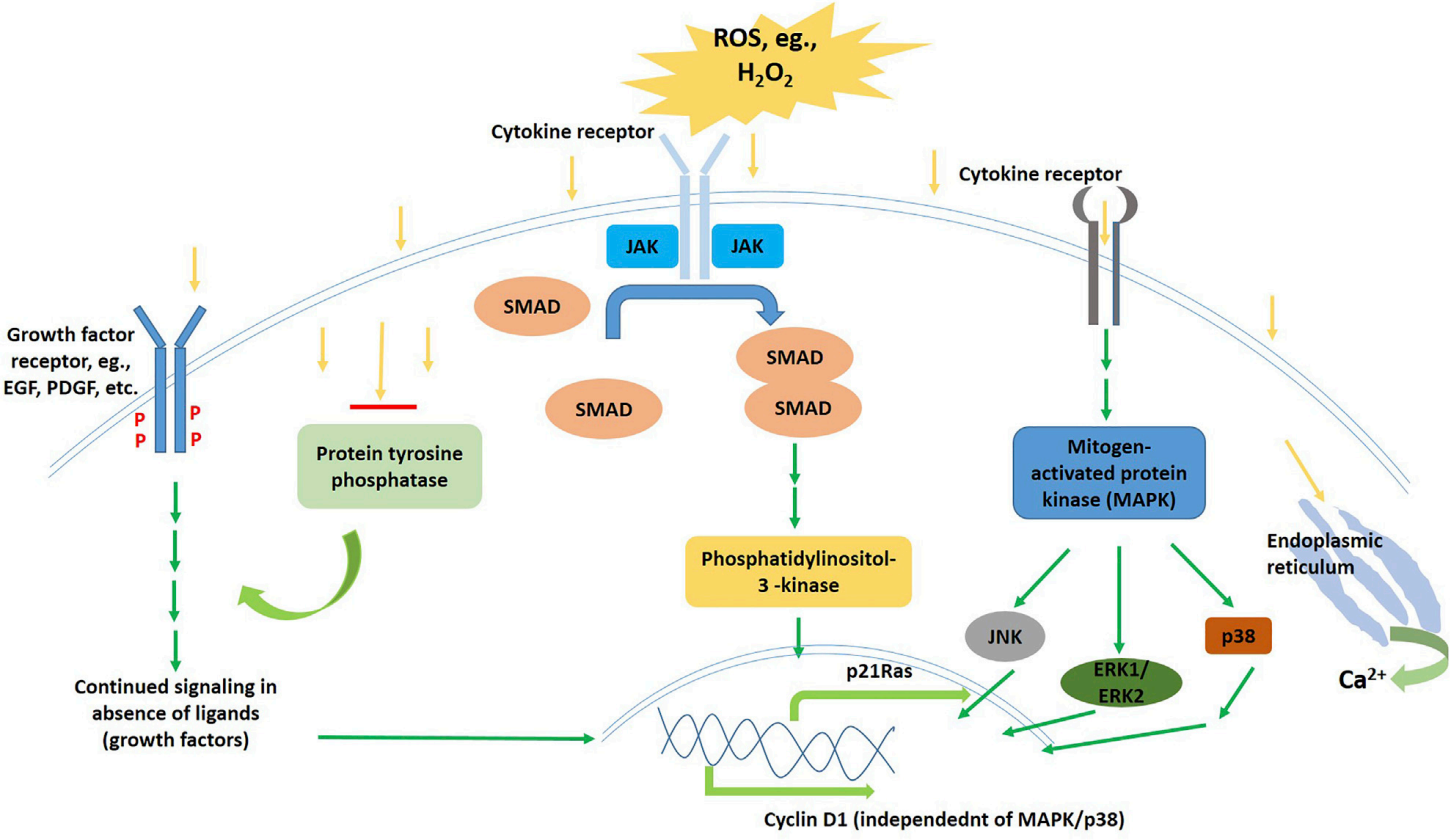
Schematic diagram of the Wnt signalling pathway in the inactive (left) and the active (right) states.

It appears that ROS affects and regulates quite a large array of signaling pathways, and this interaction might occur at various points starting from the cell-surface receptors till the nucleus (Figure 3). Growth factor receptors that commonly get activated upon ligand interaction might also get clustered and activated in the absence of ligands, sometimes under the effect of ultraviolet light, by the mediation of ROS. For example, EGF, PDGF-a, as well as PDGF-b get phosphorylated and activated by the presence of exogenous H_2_O_2_ in millimolar concentrations (Wang et al., 2004; Eisel et al., 2013; Truong and Carroll 2013; Sies and Jones 2020); apart from that, H_2_O_2_ induces PLD activation in fibroblasts and endothelial cells (Natarajan et al., 1996; Ito et al., 1997; Parinandi et al., 1999). Immediately formed ROS is the key role player in the lysophatidic acid induced activation of EGF receptors (Moolenaar et al., 2004; Hopkins et al., 2016). Knebel et al. suggested that the mechanism behind these observations might be the inhibition of RTK dephosphorylation, which might be mediated by ROS following the inactivation of protein tyrosine phoshatases that are membrane-bound (Knebel et al., 1996; Meves et al., 2001; Östman and Böhmer 2001). This phosphorylation-dephosphorylation balance gets hampered even in case of NOS as well as ROS mediated PDGF receptor activation in absence of the PDGF ligand. A separate study indicated that PLA2 might also be a target of ROS, as PLA2 mediated EGF signaling is sensitive to antioxidants (Rhee 1999).

The answer to whether or not the endogenous ROS that generate in response to growth-factor stimuli, might cause such receptor activation, needs more research work. In the same way, whether ROS during the pathophysiological state of oxidative stress is capable of activating receptors directly, or if there exist specific cytokines or growth factors that are targeted and their signaling further mediated by ROS, are also matters of further research. Phospholipid metabolites are of high importance as targets for redox signaling, and Takekoshi et al. diligently showed that when DAG was oxidized, it activated PKCs more effectively than its non-oxidized counterparts (Takekoshi et al., 2003).

Even the non-RTKs like Src and JAKs might be regulated by mainly exogenous ROS (Thannickal and Fanburg 2000; Bode et al., 2012). However, endogenous ROS might mediate the activation of JAK-STAT pathway when it is induced by PDGF (Simon et al., 1998; Simon et al., 2002).

Molecules like hydrogen peroxide (H_2_O_2_), and other ROS as well as nitrogen oxides (Nox) might induce p21Ras *via* the JAKs and Src kinases with the involvement of phosphatidylinositol 3′-kinase (Poli et al., 2004; Heneberg and Draber 2005; Monteiro et al., 2008). Intracellular Ca^2+^ concentration changes induced signaling cascades in muscle cells has also been found to be ROS-mediated. ROS might induce the release of Ca^2+^ into the sarcoplasm (Favero et al., 1995; Okabe et al., 2000; Hamilton et al., 2018). Even if these are all seemingly oxidative stress responses, nonetheless, the important role played by the ROS in induction of, and regulation of these stress-pathways cannot be disregarded, as they act as stimuli for the cascades to begin with. For the same reason, ROS affects MAPK pathways activation and cascade sustenance, as they are primarily stress-activated protein kinases (Zhang et al., 2003; Diebold and Chandel 2016). The redox regulation of these pathways, that include JNKs, ERK1/ERK2, as well as p38 MAPKs, both by exogenous as well as endogenous ROS, has been under intense research. It has also been found that redox-regulated mitogenic signaling may target, independent of the ERK/MAPK pathway, the expression of cyclin D1 (Menon 2006). Stress apoptotic responses are also found to be directly and indirectly affected by ROS (Jiang et al., 2011).

Not surprisingly, signaling cascades set off by binding of inflammatory cytokines are ROS-responsive, and this has been documented in cases of ischemia-reperfusion (Cadenas 2018) and hypoxia-reoxygenation (Adler et al., 1999; Jin and Flavell 2010; Sverrisson et al., 2015; Lu et al., 2018). The transcription factor NF-κB that regulates an array of immune and inflammatory gene responses has been considered as heavily responsive to oxidants for a long time, and as such, remarkable progress has also been made to elucidate the mechanisms by which NF-κB might get activated (Ali and Sultana 2012; Huang et al., 2016). However, as compared to the long-standing traditional viewpoint that oxidative stress is the customary mediator of varied NF-κB activators, recent updated studies have concluded that the redox-dependent activation of NF-κB is specific to the stimulus that is received, and the cell that receives the stimulus (de Bittencourt Pasquali et al., 2013). Other than that, the transcriptional complex Activator protein-1 or AP-1 has been also shown to be redox-regulated, where; both ligand-induced ROS as well as exogenous oxidants (and also some antioxidants) have been implicated in the activation of AP-1. Like AP-1, binding of other transcription factors to DNA being regulated by redox mechanisms include p53, c-Myb, Sp-1 and egr-1 (Meyer et al., 1994; Schulze-Osthoff et al., 1995; Shatrov et al., 1997).

Given the plethora of instances where ROS has been found to activate even the most basic pathways like growth-factor and cytokine signaling, as well as cell-death, mitosis, growth and stress-response mechanisms, it is implicit to check on the point as in how ROS might affect developmental signaling, especially where Wnt proteins are active.

## 2 ROS-Wnt Interplay in Developmental Signaling Networks

### 2.1 General Mechanistic Aspects

From the above information, it can be deciphered that the acceptance of ROS as signaling molecules and second messengers, which communicates messages from the extracellular milieu and generates some specific response in the cell, is concrete. Through redox modifications at mainly cysteine residues in proteins (kinases, phosphatases and transcription factors), and some other amino acids like histidine, tyrosine, and tryptophan, ROS play the role of a significant family with varied molecular diversity, that participates in almost every signaling pathway known to scientists (Barford 2004). What is important to note here is that, the redox regulation of these pathways is not entirely just ROS-mediated. Sometimes, this regulation is achieved indirectly, by interacting with antioxidants like thioredoxins (Trx) and peroxiredoxins (Prx) (Bindoli et al., 2008; Winterbourn and Hampton 2008; Hanschmann et al., 2013; Netto and Antunes 2016).

**FIGURE 4:**
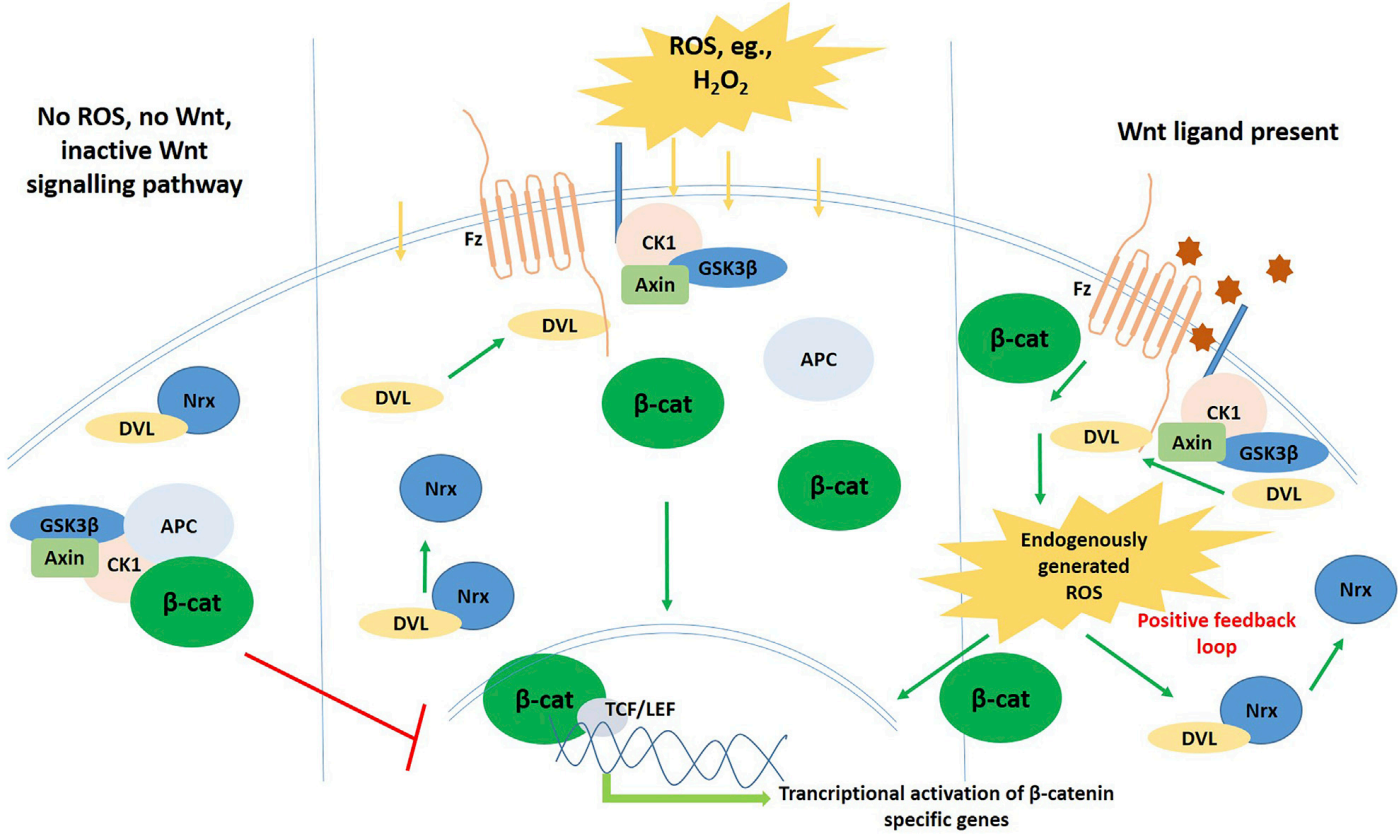
Schematic diagram showing the crosstalk and interplay of ROS with Wnt.

Now, it is also known that Wnt ligand binding to the Fz receptors stabilizes and accumulates β-catenin in the cytosol. This is achieved by the destruction of the β-catenin degradation complex, mediated by a protein named Dishevelled (Dvl). The breakthrough came when Funato et al., in 2006 discovered that ROS might be involved in the Wnt signaling mediation, and that too, *via* this protein Dvl. Nucleoredoxin (NRX), an ubiquitously expressed protein of the Trx antioxidant family, was found to directly control Dvl activity in its reduced form, by forming a complex with Dvl, thereby, keeping it inactive in the cytoplasm (Funato et al., 2006; Funato and Miki 2010). This inactive Dvl suppresses the Wnt signaling pathway; only when Dvl translocates to the plasma membrane from cytoplasm, can the Wnt signaling pathway get activated. Funato et al. showed that Dvl was freed from its inhibition complex upon treatment of culture cells with exogenous compound that is pro-oxidant in nature, and this event crucially led to the Wnt/β-catenin canonical pathway stimulation–that is the basis of an array of growth factor signaling, essential for embronic pattern formation and perinatal development of animals. They thus showed that intracellular ROS levels do positively regulate the Wnt/β-catenin pathway, by making Dvl available for transduction of the Wnt signal. This groundbreaking finding was corroborated by multiple experiments in other cells, including Xenopus embryos (Funato et al., 2008; Love et al., 2013). In these amphibian embryo models, it was found that depleting or enhancing the levels of Nrx, produced certain abnormalities in the embryo, that are specific to deregulated Wnt signaling (activation and inhibition of the pathway, respectively). It was further found that the moderately potent and generally highly scavenged ROS H_2_O_2_ oxidises Nrx, and this event releases Dvl from its inhibition, promoting the activation of the Wnt/β-catenin pathway, in the absence of Wnt ligands (Figure 4).

It was also shown by Kajla et al. that, when mouse intestinal cells were treated with Wnt, it induced the production of ROS, mediated by Nox1, *via* the activation of Vav-2, the Rac1 guanine nucleotide exchange factor (Kajla et al., 2012). Then these Nox1-generated ROS go on to oxidize and inactivate NRX, thus rescuing the cells from NRX-dependent suppression of Wnt/β-catenin signaling, by the dissociation of Dvl from NRX.

In the non-canonical pathway, Dsh (non-mammalian form of Dvl) mediates the binding of profilin to actin, by forming a complex with rac1. This binding event of profilin to actin leads to cytoskeletal restructuring, and gastrulation. Thus, indirectly, ROS mediates gastrulation *via* the non-canonical Wnt pathway (Habas et al., 2001; Andrea 2018).

Apart from the Trx family proteins, another family of peroxidase antioxidants, peroxiredoxins (Prdx) have been identified in mammals having six isoforms (known as of now), that are directly involved in H_2_O_2_ elimination, and neutralization of other pro-oxidants and ROS (Rhee and Woo 2011). Prx proteins need Trx proteins as reducing partners to function efficiently. It has been found that the Wnt/β-catenin pathway works in effecting decreased proliferation and enhanced apoptosis of colorectal cancer cells in Prdx2 knock-down models (Kim et al., 2017; Nicolussi et al., 2017; Hong et al., 2018; Chen et al., 2020).

Thus, it can be firmly suggested that ROS levels are important determinants for the functioning of Wnt pathway, and hence, must be tightly regulated to effect development correctly (Han 2015).

### 2.2 Specific Interactions and Roles

ROS has been shown to mediate cellular responses towards intra-as well as extracellular conditions, such as presence or absence of cytokines and growth factors, state of nutrient deprivation, as well as hypo- and hyperoxia, all of which in turn, regulate cell proliferation, growth, differentiation, and apoptosis (Davalli et al., 2016). The following sub-sections outline the cross-talks occurring between Wnt and ROS signaling pathways and molecules, in various aspects of embryonic developmental processes.

#### 2.2.1 Cell Death and Apoptosis

As concluded from the above information, varying concentrations of ROS elicit varying cellular effects, such as, intracellular ROS sourced from NADPH oxidase (occurring at low levels) have pro-survival effects, whereas ROS derived from mitochondria (occurring at high levels) have anti-survival effects and promote apoptosis (Angkeow et al., 2002; Deshpande and Kehrer 2006). Early embryogenic processes in mammals, especially preimplantation and early postimplantation processes have been documented to occur in highly hypoxic conditions, in an almost anaerobic environment, at only about 3–5% O_2_ conditions. Hence, it is implicated that the early embryo must be very sensitive to ROS or ROS-like exogenous factors, which might cause fatally culminating oxidative stress. High O_2_ concentrations *in vitro* embryo culture conditions is heavily detrimental to the embryological growth, and this deterioration can be reversed by treatment with free radical scavengers (Gardiner and Reed 1994). The loss of Trx gene (mediators of ROS with Wnt signaling) has been shown to affect oogenesis as well as early development of *Drosophila* embryos (Missirlis et al., 2003). The event of mouse blastocyst hatching from zona pellucida essentially requires a superoxide burst. The essential role of ROS in mediating this process is supported by the observation that various superoxide scavengers actively prevent the hatching of the blastocyst (Dumollard et al., 2006).

#### 2.2.2 Embryological Patterning

Unfit cells require to be eliminated for precise patterning of the embryo during early development. A major way to get rid of these unfit cells is by the induction of ROS and further ROS-mediated apoptosis. Akieda et al., in 2019 reported that, when Wnt/β-catenin is abnormally activated or inhibited in a mosaic manner in normal embryos, it led to activation of ROS production and further oxidation of DNA (Akieda et al., 2019). Their studies further suggest that these cells displaying unfit Wnt/β-catenin activity mediate the activation of ROS production through Smad and cadherin signaling. This was crosschecked by the finding that overexpression of antioxidants like Sephs1 or SOD1 blocked the activation of pro-apoptotic caspase-3 in artificially introduced cells where the Wnt/β-catenin pathways acts in abnormally high or low manners (unfit cells), giving concrete evidence that apoptosis of unfit cells is mediated by ROS.

The study demonstrated an interesting ‘noise-cancelling system’ model for signaling gradients that is mediated by cell-competition. During AP patterning in zebrafish embryos, extent of Wnt/β-catenin activity influences cadherin and membrane β-catenin protein level gradient formation along the AP axis. Now, when cells displaying unfit Wnt/β-catenin activity appear spontaneously in the developing embryo, it produces ample noise in the gradient of Wnt/β-catenin as well as alterations in cadherin and membrane β-catenin protein levels; this leads to a significant imbalance between the unfit and neighbouring cells based on cadherin levels. Unfit cells then activate the TGF-β-Smad signaling pathway that produces ROS, and subsequently undergo a ROS-mediated apoptosis. In this very specific ‘identify, pick and eliminate’ process, the embryonic tissues successfully eliminate noise generated in the Wnt/β-catenin gradient in order to its effect proper formation, besides a robust patterning of the embryo. This morphogen noise-cancelling system mediated by apoptosis is thought to be evolutionarily conserved, as it has been found that Wnt/β-catenin as well as other basic morphogen signaling pathways utilize similar systems. The inhibition of ROS signaling actually reduces the magnitude of physiologically appearing apoptotic cells, albeit partially, which corroborate the view that these apoptotic cell populations comprise ROS activated cells that are Wnt/β-catenin defective, besides other cells that may be potentially defective in some other morphogen-signaling systems (Akieda et al., 2019).

#### 2.2.3 Lung Development, Differentiation and Damage

As is the case with other body cells, lung cells are also lethally affected by excessive ROS accumulation within them, leading to cellular stress, damage and death. But previous studies have shown that this oxidative damage is concentration dependent. And so is the case with the expression effects ofWnt5α gene. The studies pertaining to effects of ROS during embryonic development were mainly conducted in alveolar epithelial cells (AEC IIs), as they provide the best possible environments for cellular culture and experimentation for the relevant studies. But clinical studies were also conducted to observe the effects of ROS in adult patients, for preliminary acquisition of information. Patients with acute oxygen deficiency have to undergo oxygen therapy, usually at a stable O_2_ concentration of 40%, until the gas balance is normalized in the blood. Li et al., in 2013 tried to understand the extent and manner in which ROS can cause oxygen toxicity to the lungs, and so they treated cells with O_2_ at varying concentrations at room temperature. They found that the lower O_2_ fraction (40%) had fared better in terms of survival rates and apoptosis than their higher O_2_ fraction (95%) counterparts. These good survival rates in the lower O_2_ fraction subsequently decreased with the increase in their O_2_ treatment dosage, or with increased duration of low O_2_ that they received. They went further to examine the relationship between Wnt signaling pathway and ROS in lung cells as it is known that the Wnt/β-catenin pathway plays critical roles in lung development in its early stages. Numerous factors in the pathway have been found to participate and play specific roles in the control and regulation of lung development, such as proliferation and differentiation of lung cells, formation of steric configuration of the lung, as well as specific development of the lung’s distal end. For example, Wnt5α gene is implicated to play a role in the development of lung vessels as well as the embryonic pulmonary parenchyma (Li et al., 2013). While studying the concentration and time dependent effects of ROS on lung cells, Li and his co-workers also found that after a smaller time interval, Wnt5α as well as nuclear β-catenin levels were elevated with the concurrent elevation of ROS levels in the 95% O_2_ treated group. But after a 24 h’ time interval, their expression fell to a steep low, even lower than room air treated lung cell groups.

Thus the study suggested that in alveolar epithelial cells (AEC IIs) a higher O_2_ concentration might stimulate the Wnt pathway in their early stages, thus causing premature expression of Wnt5α as well as β-catenin, both of whose levels subsided with prolonged exposure to very high concentrations of O_2_. Therefore, it was seen that Wnt pathway might function early in the development of hyperoxia-induced lung injury, which is contrary to the general understanding that Wnts help in cell growth and proliferation, and are activated under lower concentrations of oxidants like H_2_O_2_ and inactivated at higher concentrations, as has been seen in the previous sections. The authors attributed the low survival of high O_2_ fraction to oxidative damage and lung cell death. Hence, the effects of ROS mediated Wnt signaling may be diversified according to sources and cellular types, as well as the magnitude of oxidative stimulation.

#### 2.2.4 Neural Development and Differentiation, Senescence and Neuronal Damage

Rharass et al., in 2014, conducted experiments on hNPCs (human Neural Progenitor Cells) which provided concrete evidence for an endogenous ROS-mediated activation of Wnt pathway, and while it was known that changes in Ca^2+^ ion concentration are downstream effects of non-canonical Wnt signaling pathway, their study showed that the same Ca^2+^ concentration gradient acts upstream of the ROS-mediated canonical Wnt/β-catenin pathway during neuronal differentiation (Rharass et al., 2014). In hNPCs, depletion of growth factors such as EGF andbFGF acts as a molecular switch for neural differentiation, as it sets off a cascading reaction within the cell, mediated by ionic species such as Ca^2+^ and ROS. Ca^2+^ is released from the ER stores into the cytoplasm through inositol 1,4,5- triphosphate receptors, type 1 (ITPR1), once the GFs are removed, cytoplasmic Ca^2+^ enters mitochondria *via* mitochondrial calcium uniporters (MCU), thereby increasing ROS as by-products of altered ETC activity in the mitochondria. This elevated ROS then interacts with Nrx, thus, releasing the Nrx-bound Dvl, so as to stabilise β-catenin in the cytoplasm, thereby kicking off the Wnt pathway-mediated transcription of regulatory genes for growth and differentiation. This observation was cross-checked by inhibition of ITPR1 mediated Ca^2+^ efflux as well as MCU mediated Ca^2+^ influx. Besides, the experiment was repeated with the additional treatment of NAC, a highly potent ROS-quencher. In all of the above three cases, the results were consistent. It was seen that Dvl2 was no longer robustly activated, expressed by the lower levels of nuclear β-catenin accumulation, which attenuated the expression of Wnt target genes, consequently leading to an impeded differentiation of the neuronal system. It was also seen that physiological changes in ROS metabolism altered the response magnitudes of the Wnt/β-catenin signaling pathway effectors. Thus it was concluded that ROS is involved in spatio-temporally altering cellular decisions of the neuronal progenitor cells, and effected their commitment and differentiation into neurons. In embryonic development, it was an important benchmark to be known while documenting the effects of ROS and Wnt.

He et al., in 2017 discovered that mothers fed with high sucrose diet prenatally, gave birth to offsprings that had higher levels of oxidative damage in the brain, in addition to higher expression of oxidative enzymes like Nox2, and lesser expression of brain-derived neurotrophic factor (BDNF) as well as genes involved in maintenance of synaptic plasticity and dendritic arbors, like Wnt2 and Wnt3a, and NR2B (a subunit of cation-passing channels, NMDARs, in the neurons) in the hippocampus. Wnt signaling pathway down-regulation or disruption in the hippocampal region has been reported to cause cognitive decline along with neuronal loss, effects which are similar to oxidative stress induced neuronal aging and death. Thus, it can be suggested that there must be some link between the two signaling pathways in neuronal aging, about which, no concrete evidence has been found until date, and continues to remain an intense area of research (He et al., 2017).

In 2019, Diana De Luc and her co-workers found an important breakthrough in exome and genome sequencing experiments with array analysis, in a significant number of human individuals—in humans, the loss of function mutations of WDFY3 gene, lead to macrocephaly, *via* a significant role play and downregulation of the Wnt pathway, indicating the importance of the pathway in neurological and overall brain development during the embryonic stage (Le Duc et al., 2019). In the humans, the overlapping symptoms and parameters were intellectual disability, neurodevelopmental delay, macrocephaly, and related psychiatric disorders (like autism spectrum disorders/ADHD or attention deficit hyperactivity disorder). That is, this study proposed the *WDFY3* as a novel gene which is linked to mild to moderate neurodevelopmental delay in humans (initially corroborated in laboratory mice) and intellectual disability, and concluded that the mutational variants putatively causing haploinsufficiency lead to macrocephaly. The authors also proposed an opposing pathomechanism due to variants in the PH-domain of the same WDFY3, that led to microcephaly. Hence, this experiment came in as a breakthrough of how studying next-generation sequencing can actually facilitate the discovery of monogenic causes for mild to moderate neurodevelopmental delay in humans and end a long-term diagnostic blank space dilemma.

All of the above studies tried documenting the crosstalks of ROS with Wnt during neuronal development of the embryo, and as experiments, walked off with important results that worked as shoulders for future researchers to stand on.

#### 2.2.5 HSC Sustenance and Differentiation

Like in most cells, enhanced ROS has been observed to have a toxic/inhibitory effect on hematopoietic stem cells (HSCs) by impairing their functioning. β-catenin comes into the rescue and provides protection to the cells after irradiation-induced oxidative stress (Lento et al., 2014). Absence of β-catenin can result in transplantation-induced stress and ROS accumulation, and subsequently, the absolute loss of quiescent hematopoietic stem and progenitor cells (HSPCs). This transplantation-induced stress is very much suggested to be endogenous in nature (metabolic by-products of HSPCs).

For the first functional HSCs to emerge, β-catenin/canonical Wnt signaling has been implicated to be an essential event. Studies conducted by Kwarteng in 2018 indicated that mostly the canonical Wnt pathway remains active in the fetal HSPCs, but both the fetal as well as adult HSPCs are potentially able to activate the non-canonical Wnt pathways. Post-transplant, β-catenin was suggested by them to metabolically regulate and maintain short-term transplantation competitiveness of the fetal HSPCs, but the switch from fetal to the adult HSCs corresponded to a downregulation of the canonical Wnt/β-catenin pathway (Kwarteng et al., 2018). This supported the idea that, for specific HSC functioning at each developmental stage, some specific β-catenin gradient was required, and that the cells showing very high proliferation, like the leukemic cells were more β-catenin dependent than the quiescent cells. It was also suggested that β-catenin however, was not responsible for the promotion of proliferation, rather, it appeared to have a protective effect in the early stages of an emerging oxidative stress condition.

In a separate study, Boopathy et al. found that following an event of myocardial infarction, Wnt11 genes that are involved in expression of cardiogenic genes, and Notch 1, were upregulated. This further resulted in the elevation of H_2_O_2_ levels (Boopathy et al., 2014). These results mirrored those that were obtained by other researchers, that Wnt11 genetic expression increased in Endothelial stem cells along with mouse bone-marrow mononuclear cells, following an event of cardiomyogenic differentiation of them induced by oxidative stress injury (Caliceti et al., 2014).

#### 2.2.6 Vascular Differentiation

The canonical Wnt signaling pathway has been found to play an essential role in maintenance of vasculature as well as its development. Wnt2-deficient experimental mice displayed multiple vascular aberrations, including defects in the placental vasculature (Wang et al., 2007). Also, mice in which the Fz5 (Wnt receptor) gene was knocked-out, showed serious defects in angiogenesis of the yolk sac, and died *in utero* (Goodwin 2004). Vascular endothelial cells showing defects in the β-catenin gene were found to show significant abnormal vascular patterning in addition to increased fragility of the vasculature (Valerius 2012; Fritz et al., 2014).

Dishevelled (Dvl), besides being an integral adaptor protein for stabilizing cytoplasmic β-catenin and efficient transduction of the Wnt signal, is also a Notch signaling inhibitor. Therefore, Dvl plays a key role in making decisions pertaining to cellular fate, especially in cases where Wnt and Notch have antagonistic effects (Wharton 2003; VanHook 2012).

During angiogenesis, the expression of an array of Wnt ligands is upregulated by a profribrotic and proosteogenic transcription factor named Msx2, and this protein has been also found to enhance canonical Wnt signaling in aorta (Shao et al., 2007). TNFα has been found to induce these Msx2-Wnt signaling pathways in arterial myofibroblast, acting *via* the TNFR1 receptor (tumor necrosis factor receptor 1) (Lai et al., 2012). Studies of gene expression conducted *in vitro* as well as *in vivo*, concluded that inhibition of ROS derived from Nox/mitochondrial activities, reduced the induction of Msx2 by TNFα. Concurrently, the levels of Wnt7b as well as β-catenin were also found to be diminished, which led to the establishment of the idea that in myofibroblasts, ROS metabolism contributes to the induction of TNFα-Msx2-Wnt signaling cascade, *via* TNFR1 (Cheng et al., 2013).

From various studies, it has been found that ROS-modulated signaling of Wnt and Notch regulates the development of vasculature in a variety of aspects, ranging from stem cell differentiation to vascular cell migration, like VEGF signaling, angiogenesis, and recruitment of cardiac as well as endothelial progenitor cells (Marinou et al., 2012) (Caliceti et al., 2014).

#### 2.2.7 Nephrological Development

It has been documented by multiple studies on *Xenopus laevis* embryos that Wnt signaling pathways including both canonical as well as non-canonical, are indispensable regulators of pronephos development (Pulkkinen et al., 2008). The Wnt/β-catenin canonical pathway directs the majority of kidney development, and inhibitions or aberrations in this cascade might lead to significant alterations and abnormalities in pronephric tubules, glomus, and duct, in addition to overall reduction in the expression of pronesphros-specific markers (Halt and Vainio 2014). The β-catenin independent non-canonical cascade is especially involved in the formation of proximal tubules during the process of pronephrogenesis (Iglesias et al., 2007).

Just as is the case with other types of body cells, uncontrolled elevation in ROS levels leads to cellular damage. When ROS is allowed to increase unhindered during the embryonic period, massive ciliary defects are observed, leading to severe malformations of the pronephros, whereas, in the entire perinatal period following kidney development, as well as in adult stage too, elevated ROS is associated with severe kidney damage (Benzing et al., 2007). In both the fetal as well as adult cases, Prdx1 overexpression is able to significantly inhibit the effects resulting from increased ROS, thereby suggesting that Prdx1 regulates ROS production, and has a protective role for the tissues, saving them from the induction and effects of oxidative damage (Agborbesong et al., 2022).

Chae et al., in 2017 studied how these two seemingly opposite effects are modulated by the two pathways by experimenting on MDCK cells, given the established fact that healthy and normal primary cilia are required for both the canonical and non-canonical Wnt pathways to be mediated effectively (Chae et al., 2017). They found that Prdx1 was the key savior here, which mediates the genesis and development of normal cilia, by inhibiting ROS production, and thereby allowing Wnt signals to be transduced unhinhered, leading to complete and robust development of the pronephros. It was cross-checked by inhibiting Prdx1 in these cells led to significant lowering in the number of cells that had primary cilia, thus suggesting that Prdx1 was absolutely essential for primary cilia development and regulates their formation. In addition to that, they also showed in *Xenopus laevis* embryo cells treated with H_2_O_2_ that, ROS overproduction led to an inhibition in the formation of proximal tubules. But Prdx1 overexpression was able to rescue and abate this abnormal phenotype. Thus it was concluded that Prdx1 regulated the Wnt-mediated pronephros development, by keeping ROS levels in check.

In case of embryonic kidney cells of humans, nuclear β-catenin amount, and subsequently, TCF/LEF-dependent transcription of the Wnt-targeted genes are decreased by H_2_O_2_ treatment. Interestingly, Dvl2 overexpression mitigated the downregulation of β-catenin induced by H_2_O_2_ treatment, and hence, it was suggested that H_2_O_2_ negatively modulated the Wnt signaling pathway in humans, by downregulating β-catenin (Chae et al., 2017).

#### 2.2.8 Extraembryonic Endoderm Formation and Differentiation

One of the very first cellular fate determining decisions taken in the developing embryo is, differentiation of the primitive endoderm from the cells belonging to the inner cell mass. Hwang et al., in 2011, treated F9 teratocarcinoma cells (mouse embryonic carcinoma) with retinoic acid (RA), which was known to initiate differentiation of the extraembryonic endoderm *in vitro*. This differentiation is known to be executed by many pathways, including both the canonical as well as non-canonical Wnt signaling pathways, and is associated with an epithelial to mesenchymal transition (EMT). Hwang and his co-workers found that, this RA-mediated differentiation also concomitantly showed a sustained increase in the levels of ROS, which by then were already known to positively influence the Wnt signaling pathway. They then hypothesized that treating F9 cells with H_2_O_2_ could activate the Wnt signaling pathway even in the absence of Wnt ligands, such that the extraembryonic endoderm is formed, and successfully tested it, to find that H_2_O_2_ treatment indeed brought about the appearance of the markers of primitive endoderm–morphological, molecular, as well as biochemical ones. They also found the canonical Wnt/β-catenin signaling to have been activated, as well as the expression of Nox4 to have been elevated. All these RA-mediated primitive endoderm differentiation markers were inhibited when the F9 cells were pretreated with antioxidants like NAC, or Nox inhibitors like DPI, prior to RA treatment (Hwang et al., 2011). Thus, their findings paved way for the idea that ROS signaling does indeed cross paths with the Wnt/β-catenin cell signaling pathway in the developing mouse embryo, so as to positively regulate in it, the formation of the extraembryonic or primitive endoderm.

Nrx, as well as Trx, play important roles in embrogenesis and tissue patterning, which can be suggested from the fact that Trx1 as well as Nrx knockout mice pups either suffer lethality during implantation, or if alive with abnormalities as foetuses, they die soon around birth (Hwang 2014). Similar case occurs with Dvl knockout mice pups, which suffer from embryonic lethality (van Amerongen and Berns 2006; Wynshaw-Boris 2012).

Sandieson et al., in 2014, treated F9 cells with H_2_O_2_ as well as performed Wnt6 peconditioning, and in both cases, they obtained results that mimicked that of Hwang et al., that is, similar diffentiation occurred, accompanied by ROS generation, as happens in case of RA-induced differentiation. They further found that the redox state of Nrx, as expected, dictates the formation of extraembryonic endoderm. Nrx bound to Dvl2 in the undifferentiated F9 cells, and H_2_O_2_ treatment rescued this bondage, in line with other studies. When Sandieson and his co-workers depleted Nrx levels, similar morphological changes were observed as in the case of RA-induced differentiation, and when they increased the activity of Protein Kinase A, the primitive endoderm cells were further induced to parietal endoderm cells. In line with other studies, this depletion of Nrx also led to an increased transcription of TCF/LEF targeted genes, which indicated the canonical Wnt signaling to be in action (Sandieson et al., 2014). Their studies thus corroborated the idea that Nrx inhibits Wnt signaling in F9 cells, keeping them off; after the reception of Wnt ligands or ROS, this constraint is lifted. Unbound Dvl2 then positions itself to conduct the Wnt signaling pathway required for the formation of primitive endoderm.

#### 2.2.9 Cardiac Differentiation and Protection

The prosurvival mTOR signaling pathway, and the Wnt signaling pathway, mediate cardioprotection *via* ROS, by promoting Akt activation and inhibiting GSK3-β (Yao et al., 2014). Any disruption in the Wnt pathway modulates this protective loop and activates GSK3-β, which is a part of the β-catenin degradation complex (Vigneron et al., 2011). Ischemic preconditioning, which ensures cardioprotection, is abolished with secreted frizzled protein 1 (sFRP1) overexpression, as, sRFP1, which is a Wnt/Frizzled pathway antagonist, decreases GSK3-β′s phosphorylation and inhibition (Barandon et al., 2005). Studies by Coant et al. showed that Nox1 directly or indirectly redox-regulates the Wnt and PI3K/Akt signaling pathways, thus enhancing the nuclear translocation of β-catenin, as well as the activation of Notch1, a member of the Notch superfamily, which mediates another important embryonic cell signaling pathway. Loss of Nox1 and its redox-regulation resulted in the activity of PTEN to rise, and inhibited the Akt signaling pathway, in addition to Notch1 as well as Wnt/β-catenin signaling inhibition (Coant et al., 2010) (Zhang et al., 2015).

Funato et al., in 2010, reported that Nrx plays an interesting role in cardiac and its precursor cells. They observed that Nrx deficient mice showed cardiovascular defects. On studying, they found that in cardiac cells, Kelch-like 12 proteins are highly expressed, which target Dvl for ubiquitination. Nrx in cardiomyocytes acts antagonistically to Klhl-12, and thereby acts as Dvl stabilizer. Nrx binding to Dvl releases Dvl from Klhl-12; Dvl is thus no longer ubiquitinated, and thus, Nrx maintains a robust pool of Dvl in the cytoplasm to work when the cells receive Wnt signals (Funato and Miki 2010).

#### 2.2.10 Fibrogenic Differentiation: Osteogenesis, Adipogenesis, and Chondrogenesis

Wnt plays the role of a molecular switch in case of osteogenic/adipogenic differentiation (Benayahu et al., 2019). The Wnt/β-catenin canonical pathway favors the activity of osteoclasts and osteoblasts and thus, positively regulates it. β-catenin has been found to induce important signals for the initiation of osteogenesis, and inactivation of these initiation signals under specific conditions has been shown to lead to the conversion of osteoblasts into chondrocytes, thereby delaying the mineralization of the skeleton. On the contrary, adipogenesis is suppressed by Wnt, by the reduction in expression of key molecular regulators of adipogenesis, like PPARg mRNAs and C/EBPa (Mathieu and Loboa 2012; Tian et al., 2020).

In conditions of oxidative stress, this fine-tuned regulation is disrupted, and the entire cascade takes a different path. H_2_O_2_ suppresses TCF-mediated transcription and affects osteoblastogenesis adversely, but β-catenin overexpression mitigates this suppression. The reason why osteogenesis diminishes with age has also been traced back to ROS levels in the body, which increase with age, and then consequently decrease Wnt target genes’ expression, like Opg and Axin2. Thus, the osteoinductive effect that Wnt displays, is inhibited by ROS, even when in normal conditions, this pathway is indispensable for the osteogenesis process to be induced and conducted (Atashi et al., 2015).

Increase in oxidative stress along with a decrease in production of growth factors are symptoms associated with bone involution processes, like that happens during decreased bone formation, as well as with increased adiposity of the bone marrow. During these times, FOXO family of transcription factors get activated, which govern the primary defense mechanisms against oxidative stress in the cell. If these cells are osteoblast progenitors, FOXOs under oxidative stress, associate in an increased magnitude with β-catenin, get translocated to the nucleus, and performs transcriptive induction of antioxidant target genes, as well as those associated with cell longevity, dormancy, and cell cycle arrest. FOXOs are known to antagonize Wnt signaling in osteoblasts and osteoblast progenitor cells, thereby repressing osteogenic differentiation (Almeida et al., 2007).

Thus, it can be concluded that osteogenic, chondrogenic, and myogenic differentiation from mesenchymal stem cells are all promoted by the Wnt signaling pathway, whereas, the same pathway suppresses adipogenic differentiation from preadipocyte cells. Under normal conditions, FOXO negatively regulates adipogenesis. However, under the influence of an oxidative environment, β-catenin, the integral cascade factor of Wnt signaling pathway gets diverted to the FOXO signaling pathway, and Wnt signaling is suppressed, and thus adipogeneis is then favored (Iyer et al., 2013; Ma et al., 2020).

#### 2.2.11 Cellular Senescence

**FIGURE 5:**
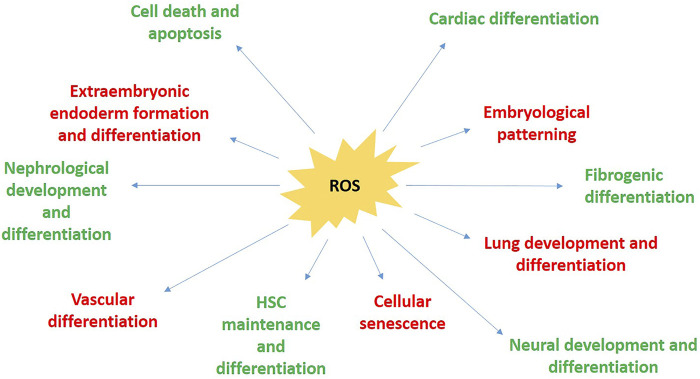
The different areas afflicted by the implication of ROS during perinatal development.

It is a well-established fact that ROS levels, when elevated chronically, lead to accumulated effects of oxidative-stress mediated cell damage, further leading to senescence and cell death, or transformation to malignancy (Azad et al., 2009). But in recent times it was also found that continuous exposure of fibroblasts can also set off events leading to senescence in animals as well as cells in culture (Marchi et al., 2012). Yoon et al., in 2010 tried studying the links if any, between the two pathways. They treated mouse embryonic fibroblasts with Wnt3A over an extended period (2 weeks) at low O_2_ levels (physiological concentration of 3%, rather than 20% that is used in standard tissue culture, to avoid interference and oxidative stress from external atmospheric oxygen and its induced replicative senescence) such that senescence in these culture cells could be delayed (Yoon et al., 2010). They observed that even in those extended periods of low O_2_ conditions, the cells showed enhanced levels of oxidation markers (8-oxoguanine), which they obviously concluded, to be coming from elevation of endogenous ROS from mitochondria, thereby shifting these Wnt3A-treated cells into oxidative stress. Concomitantly, they observed decrease in proliferation, along with enhanced levels of senescence markers (β-galacosidase staining). They re-checked their experimental findings by repeating the same experiments with the addition of NAC (N-acetyl Cysteine), a molecule which effectively normalised the aberrant parameters. Wnt mediated mitochondrial biogenesis was still underway in these NAC-treated cells, confirming the established idea that Wnt directly activated mitochondrial biogenetic process rather than as a secondary response to elevated ROS levels. But in contrast to that, elevated ROS levels were essentially required to induce Wnt-mediated senescence, thereby suggesting an upstream role of Wnt in induction of replicative senescence in these MEFs, by increment of mitochondrial biogenesis, which elevates the cellular ROS levels, and its consequential ROS-mediated cellular damage (Yoon et al., 2010) (Figure 5).

## Conclusion

A great deal of research has gone into studying the canonical Wnt/β-catenin signaling pathway in the deepest details, and most, if not all of them have suggested that it plays a key role to induce, manage, execute, and regulate the various events that are required for normal development of the embryo, as well as cellular maintenance in adult life. Because of its main engagement in the growth, proliferation, development and differentiation processes of the cells and their progenitors, the Wnt signaling pathway has also been implicated to be one of the root causes of several human diseases, including neonatal and congenital conditions, and cancer. Stem cell research for therapy has made its foray into the minutest details of this pathway in the past few years, given its important role in maintaining all kinds of adult, embryonic, adult, as well as cancer stem cells. Hence it becomes an important task to find and learn about the regulatory mechanisms of the Wnt pathway under normal conditions, and how aberrations in these cascades can be mitigated, such that any deadly disease state can be prevented from onset or progression.

ROS on the other hand have been for long, deemed as toxic molecules, leading to various diseases across all the organs of the body, leading to an overall outcome of cell damage and death in most, if not all cases. But with the broadening of research, they have increasingly come to be recognized as essential regulators of a plethora of biological functions and modulating various states of pathophysiology. They have been found to act at various nodes of signaling cascades, activating or inhibiting them, to induce significant toxic or pro-survival effects. It has been convincingly established that the oxidants could be regulated at their production source, and could be very specific in what their effects are. That the earliest organisms like bacteria, and plants heavily rely on significant aspects of oxidant signaling mechanisms and pathways, suggests that oxidant signaling mechanisms are in all probabilities, ancient, and might have been evolutionarily conserved.

We have discussed here a significant role of ROS–in mediating the Wnt/β-catenin signaling cascade and its modulation. We have henceforth concluded that clear and correct functioning and cross-talk of these two signaling pathways of ROS and Wnt are essential for appropriately and robustly deciding the cell-fate. Whereas we have mostly focused on the term ‘signaling cascades’, we must keep this in mind that the cell is an active network of multiple signaling cascades working in tandem, to bring about any particular effect. And these networks are active every moment that we are living, to facilitate our effective and healthy life. Hence, more research on the ROS modulation of these cell-fate deciding signaling pathways like Wnt, as well as Notch, Hedgehog, etc., along with the cross-talks going on in these networks, would provide important clues for strategizing clinical practices, therapies, and drug discovery, for targeting the diseases that might affect foetuses and neonates during the perinatal period, as well as for later diseases like aging and amyloid induced neurodegeneration, cardiovascular diseases, liver as well as kidney ailments, and cancer.
